# Preparation and Characterization of Chitosan—Agarose Composite Films

**DOI:** 10.3390/ma9100816

**Published:** 2016-09-30

**Authors:** Zhang Hu, Pengzhi Hong, Mingneng Liao, Songzhi Kong, Na Huang, Chunyan Ou, Sidong Li

**Affiliations:** 1 Department of Chemistry, College of Science, Guangdong Ocean University, Zhanjiang 524088, China; gdoulmn@163.com (M.L.); kongsongzhi@126.com (S.K.); 1138354521@qq.com (N.H.); 2 College of Food Science and Technology, Guangdong Ocean University, Zhanjiang 524088, China; hongpengzhi@126.com; 3 School of Pharmacy, Nanjing University of Chinese Medicine, Nanjing 210023, China; ocy545184@163.com

**Keywords:** chitosan, agarose, composite films, properties

## Abstract

Nowadays, there is a growing interest to develop biodegradable functional composite materials for food packaging and biomedicine applications from renewable sources. Some composite films were prepared by the casting method using chitosan (CS) and agarose (AG) in different mass ratios. The composite films were analyzed for physical-chemical-mechanical properties including tensile strength (TS), elongation-at-break (EB), water vapor transmission rate (WVTR), swelling ratio, Fourier-transform infrared spectroscopy, and morphology observations. The antibacterial properties of the composite films were also evaluated. The obtained results reveal that an addition of AG in varied proportions to a CS solution leads to an enhancement of the composite film’s tensile strength, elongation-at-break, and water vapor transmission rate. The composite film with an agarose mass concentration of 60% was of the highest water uptake capacity. These improvements can be explained by the chemical structures of the new composite films, which contain hydrogen bonding interactions between the chitosan and agarose as shown by Fourier-transform infrared spectroscopy (FTIR) analysis and the micro-pore structures as observed with optical microscopes and scanning electron microscopy (SEM). The antibacterial results demonstrated that the films with agarose mass concentrations ranging from 0% to 60% possessed antibacterial properties. These results indicate that these composite films, especially the composite film with an agarose mass concentration of 60%, exhibit excellent potential to be used in food packaging and biomedical materials.

## 1. Introduction

Because traditional food packaging materials cause so many environmental problems, much attention has recently been paid to biodegradable materials from renewable sources, particularly those with antibacterial properties [1,2]. Biological polymer films can be used as a protective coating to decrease the environmental impact on food and maintain food quality. Coatings and films have been fabricated with biological molecules such as polysaccharides, proteins, lipids, or combinations of the above due to their high film-forming ability.

**Figure 1:**
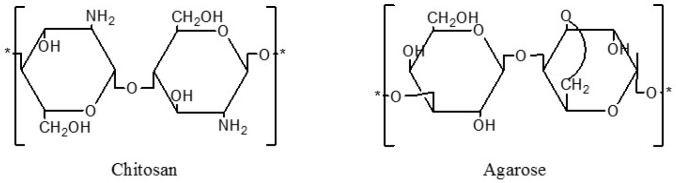
Chemical structures of chitosan and agarose.

Chitosan (CS), a naturally occurring linear cationic polysaccharide (Figure 1), has received much interest recently for its non-toxicity, biocompatibility, biodegradability, and bioactivity, and it has been extensively applied in agriculture, biotechnology, biomedicine, and the food industry [3,4,5]. Particularly noteworthy is that chitosan is a promising material for packaging films because of its film-forming properties and strong, broad-spectrum antibacterial and antifungal capabilities [6,7]. Although it has a broad spectrum of antimicrobial activity, chitosan exhibits a different kind of inhibitory efficiency against the different target organisms. Some studies have shown that chitosan generally shows stronger bactericidal effects against Gram-positive bacteria than does Gram-negative bacteria, perhaps as a consequence of the Gram-negative outer membrane barrier [8,9,10]. No et al. [9] reported that *Staphylococcus aureus* (*S. aureus*) is almost or completely inhibited, and *Escherichia coli* (*E. coli*) is slightly inhibited by 0.1% chitosan treatment.

Agarose (AG), which can be extracted from marine red algae, is a biocompatible linear polysaccharide (Figure 1). Owing to its especially hydrophilic and macroporous structure, agarose has a distinct ability to form a thermally reversible gel. The mechanical properties of agarose are similar to those of tissues and can be easily modulated by changing its content. Due to its renewability, biodegradability, and strong gelling power, agarose has been regarded as a strong potential candidate for use in biomaterials [11,12,13].

To take advantage of each individual component, material blending is an effective approach to obtain materials with ideal functional properties, and great progress has been made in the areas of food packaging and biomedical materials [14,15,16,17]. However, few studies have reported the combination of chitosan with agarose in films or coatings. In the authors’ previous paper [18], chitosan–agarose composite microspheres were successfully prepared for berbamine delivery. It was found that drug adsorption and release efficacies of chitosan microspheres were improved by the introduction of agarose. In order to obtain composite films with excellent properties for food packaging or biomedical applications, the objective of the present work was to fabricate chitosan–agarose blend films and to elaborate the effect of compositions on the performance of these composite films.

## 2. Results and Discussion

Some glossy and elastic films were successfully peeled from the Teflon-coated glass dishes. They appeared to be cream-colored and did not easily break. 

### 2.1. Mechanical Properties

**Figure 2:**
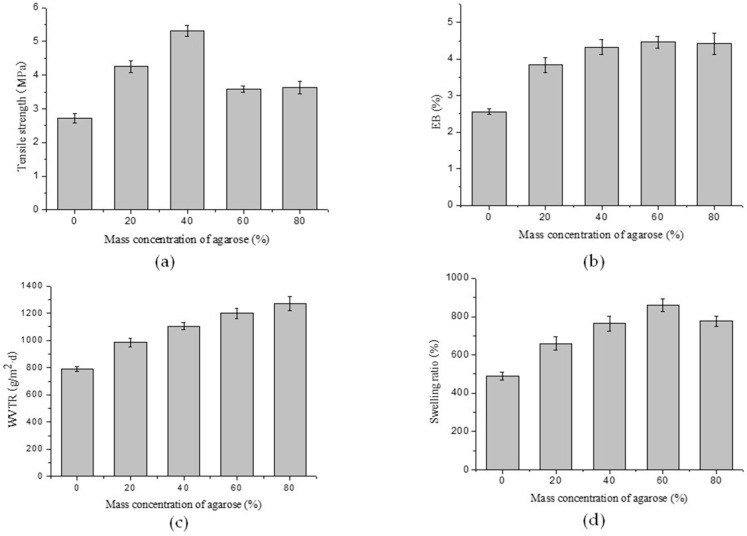
Effects of agarose mass ratios on (**a**) tensile strength; (**b**) elongation-at-break; (**c**) water vapor transmission rate; and (**d**) swelling ratios of the composite films.

Films that are used for packaging are generally required to withstand external stress while maintaining their integrity and barrier properties. This requires flexibility and good mechanical properties. The tensile strength (TS) of chitosan–agarose composite films with different mass ratios was measured, and the results showed that TS values of the films increased when AG was incorporated into CS films. The TS values of the films markedly increased from 2.72 to 5.31 MPa as the AG concentration rose from 0% to 40%. The tensile strength of the composite film with an agarose mass concentration of 40% was approximately double that of the chitosan film without any agarose. However, the TS values of the films slightly decreased when the mass concentration of AG increased from 60% to 80% (Figure 2a). These results might indicate that the formation of intermolecular hydrogen bonds between the NH_2_− in the CS and the OH− in the AG led to the increase of the TS value of the films. As the AG concentration exceeded 40%, the decrease in TS may have resulted from a phase separation between chitosan and agarose for more hydrogen bonds forming among intramolecules rather than intermolecules. These results are similar to those reported in other studies [19].

As seen in Figure 2b, the elongation-at-break (EB) property of the composite films was enhanced by the introduction of AG into the CS film. However, the addition of too much AG did not significantly increase the flexibility of the composite films. The composite films were superior in mechanical properties compared with the previously studied films that used chitosan alone [20]. As a consequence, the experimental techniques adopted in this work not only successfully fabricated CS–AG composite films, but also enhanced the mechanical properties of the CS–AG composite films significantly.

### 2.2. Water Vapor Transmission Rate (WVTR)

The water vapor permeability of a film in food packaging plays an important role in food deterioration, and it is closely related to the WVTR of the film. To evaluate the influence of AG on the WVTR values of the composite films, CS films with different mass concentrations of AG ranging from 0% to 80% were investigated, and the results showed that the WVTR values of the composite films increased as AG was added in greater concentrations (Figure 2c). The results could be attributed to the exceptional gel properties of agarose. A gel with three-dimensional porous network structures forms, providing a good environment for water vapor transmission, when agarose is solubilized in water.

### 2.3. Swelling Test

The results obtained for swelling ability are shown in Figure 2d. The data demonstrates that the chitosan films prepared without agarose (0% AG) and with agarose (20%–80% AG) were all of high water uptake capacity, but those with agarose performed better. The high water uptake capacity of the composite films could be attributed to the hydroxyl, amino, and carboxyl hydrophilic groups that exist in chitosan and agarose [21]. A different trend was observed when the swelling results were compared to the WVTR results. The chitosan film without agarose had the lowest swelling ratio, while the composite film with an agarose mass concentration of 60% showed the highest water uptake capacity. These behaviors may be explained by the different compositions and special three-dimensional structures of different films.

### 2.4. Antibacterial Properties

The antibacterial results of the composite films with different agarose contents against *S. aureus* and *E. coli* are listed in Table 1. As can be seen from Table 1, the antibacterial zone areas of the films without agarose were the largest. It indicated that the films composed of chitosan alone showed excellent antibacterial activity against both *S. aureus* and *E. coli*. It was observed that the antibacterial zone areas of the composite films with agarose mass concentrations from 20% to 60% against *S. aureus* were also relatively large. In contrast, those of the composite films against *E. coli* were dramatically decreased. This demonstrates that chitosan had stronger antibacterial effects for Gram-positive bacteria than Gram-negative bacteria. The results are consistent with previous reports [9,22], which may be due to the different chemical structures and compositions of the cell walls between *S. aureus* and *E. coli*. As for the composite films with an agarose mass concentration of 80%, the antibacterial zone areas were the smallest. This proved that the composite films with 80% agarose contents only slightly inhibited the growth of *S. aureus* and *E. coli*, which is probably because the high concentration of agarose led to the aggregation of chitosan, resulting in phase separation, and then affected their antibacterial activity. The results of the antibacterial test suggested that the films with agarose mass concentrations ranging from 0% to 60% had some abilities against Gram-positive and Gram-negative bacteria, which rendered them promising for food packaging and biomedical applications.

### 2.5. FTIR-ATR Spectroscopy

**Figure 3:**
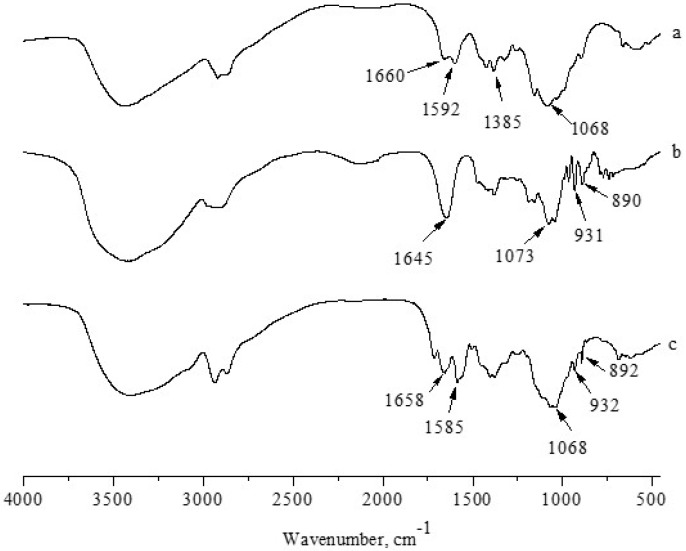
FTIR-ATR spectra of (**a**) chitosan; (**b**) agarose; and (**c**) chitosan–agarose composite film with an agarose mass concentration of 60%.

Fourier-transform infrared spectroscopy (FTIR spectroscopy) is widely applied to identify whether a certain group or chemical bond in a molecule exists or not according to the unique energy absorption. Figure 3 shows the FTIR spectra of chitosan, agarose, and chitosan–agarose composite films with an agarose mass concentration of 60%. The spectrum of chitosan film is similar to previous reports in the literature [23], and the characteristic bands of chitosan are clearly identified. The broad absorption band between 3600 and 3000 cm^−1^ could be attributed to the –OH and –NH stretching vibrations, the absorption bands at 1660, 1592, and 1385 cm^−1^ are respectively ascribed to the amide I, II and III bands, and the absorption band at 1068 cm^−1^ is attributed to the C–O stretching vibrations. In the spectrum for agarose film, the absorption band at 1645 cm^−1^ is ascribed to O–H bending, at 1073 cm^−1^, attributed to the C–O stretching vibrations. The characteristic absorption bands of 3,6-anhydrogalactose and the C–H bending vibrations of anomeric carbon appeared at 931 and 890 cm^−1^, respectively [24]. When different materials are mixed together, the changes in characteristic peaks of the infrared spectrum can reflect whether there are chemical interactions between them. In the spectrum of the composite film with agarose mass concentration of 60%, the amide II band of chitosan shifted from 1592 to 1585 cm^−1^. Obviously, the shift of the amide band of chitosan to lower frequencies resulted from the addition of agarose, indicating that the presence of agarose strengthened the hydrogen bond interactions between molecules. The difference in the composition of the films could be distinguished by the spectral shifts of the amide bands because the changes in the hydrogen bond strength are related to the concentration of the substances. In addition, the absorption bands at 1068 and 1073 cm^−1^ associated with C–O stretching joined to become one single peak, suggesting a presence of many hydrogen bonds and micro-phase separation.

### 2.6. Morphology Studies

**Figure 4:**
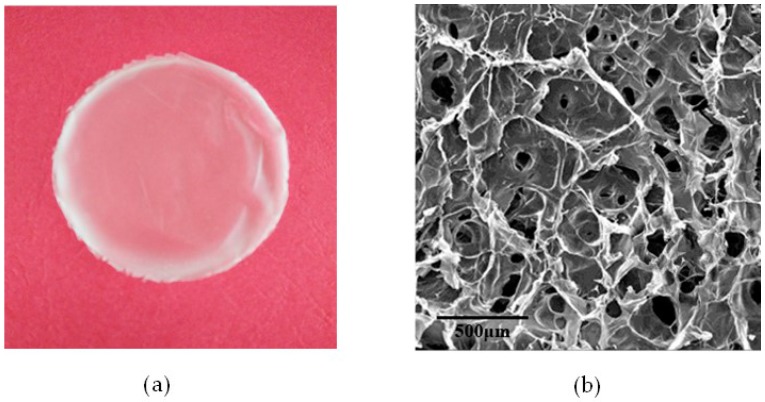
The (**a**) optical microscopy and (**b**) SEM micrographs of the composite film with an agarose mass concentration of 60%.

The chitosan–agarose composite film with an agarose mass concentration of 60% was selected for morphology studies. The surface morphologies of the film were observed with a digital camera and scanning electron microscopy (SEM). The results are shown in Figure 4. In Figure 4a, which was obtained by a digital camera, the composite film is translucent, glossy and elastic. As shown in Figure 4b obtained by SEM, the composite film is found to be highly porous. The pore morphology is between oval and polygonal. The size and density of the holes is not consistent. The formation of the holes is partly due to the rupture of the pore walls between the gaps left by the ice sublimation in the frozen composite film during the vacuum drying process. Micro-phase separation also occurred because of the different degrees of agglomeration in the blends. The micro-pore structures of the composite film were of great benefit to the air and moisture permeability, and cell regeneration and reproduction, evidencing this film’s great development prospects in the field of biomedical materials and food packaging.

## 3. Materials and Methods

### 3.1. Materials

Chitosan (CS) with a viscosity average Mw of 1.0 × 10^5^ and a degree of deacetylation of more than 85% was purchased from Greenbird Sci-Tech Development Corporation (Shanghai, China). The agarose used was provided free of cost from Taixing Bio-Technology Limited Corporation (Lianjiang, China). The acetic acid was of analytical grade. 

### 3.2. Film Preparation

The films were fabricated by the casting method [25]. Solutions of chitosan (1.5%, w/v) were prepared by dispersing 1.5 g of chitosan in a 1% (v/v) acetic acid solution and stirring for 12 h at room temperature. Solutions of agarose (1.5%, w/v) were prepared with hot distilled water stirring for 0.5 h. Chitosan–agarose solutions were produced in mixtures of chitosan and agarose with agarose mass concentrations of 0%, 20%, 40%, 60%, and 80% at 60 °C while stirring. The mixture solutions were poured into level Teflon-coated glass dishes and dried at 60 °C. Once the films were formed, they were transferred into a refrigerator to freeze and then freeze-dried for 8 h. The dried films were peeled off of the dishes and placed into a desiccator with 57% relative humidity (saturated solution of sodium bromide) for use.

### 3.3. Mechanical Properties

A vernier caliper with an accuracy of 0.02 mm was used to measure the thickness of the films. Six different regions were measured for each film. The average thicknesses of the films at different concentrations were 0.21 mm (0%), 0.15 mm (20%), 0.14 mm (40%), 0.23 mm (60%), and 0.19 mm (80%). The average thickness was used to calculate the tensile strength of the films. The tensile strength (TS) and elongation-at-break (EB) were measured by a Texture Analyzer using rectangular samples (80 mm × 25 mm) according to the reference method [26]. TS and EB were calculated according to Equations (1) and (2), respectively. The TS and EB tests were repeated five times for each type of film.

(1) $$\mathrm{TS}=\frac{S_{m}}{L\times W}\times 100\%,\;\mathrm{and}$$

(2) $$\mathrm{EB}=\frac{D_{b}-D_{i}}{D_{i}}\times 100\%,$$

where TS is the tensile strength in MPa; S_m_ is the maximum value of strength at break in N; L and W are the thickness and width of the sample, respectively, in mm; EB is the value of elongation-at-break in %; D_i_ is the original length between the two grips (50 mm); and D_b_ is the length between the two grips right before the break of each sample in mm.

### 3.4. Water Vapor Transmission Rate (WVTR)

WVTR (g·m^−2^·d^−1^) was gravimetrically determined using a method described by Leceta et al. [2] with a few modifications. Glass cups containing 100 mL of distilled water were sealed securely with the films, and their weights were recorded as W_b_. The cups were placed in an environment with a humidity of 65% at room temperature. A fan in the environment at a velocity of approximately 100 rpm was used to move the air over the surface of the films. The weights of the cups were recorded as W_a_ after keeping for one day. WVTR (g·m^−2^·d^−1^) was calculated according to Equation (3):

(3) $$\mathrm{WVTR}=\frac{W_{b}-W_{a}}{T\times A}\times 100\%,$$

where W_b_ is the weight of the testing cups before timing the experiment in g; W_a_ is the weight of the testing cups after timing the experiment for one day in g; T is the testing time in h; and A is the testing area of the films in m^2^.

### 3.5. Swelling Test

The swelling test was carried out with reference to a previously reported method [27] with a few modifications as follows. The film would be weighed (W_0_) first, then immersed in 100 mL of distilled water and placed at room temperature for 24 h. The swelling film was taken out from the solution, and the surface water on the film was sucked out by the filter paper and subsequently weighted (W_t_). The swelling ratio (%) was calculated according to Equation (4):

(4) $$\mathrm{Swelling}\;\mathrm{ratio}\;(\%)=\frac{W_{t}-W_{0}}{W_{0}}\times 100\%,$$

where W_0_ and W_t_ are the weights of the films before and after swelling, respectively, in g. 

### 3.6. Antibacterial Test

*S. aureus* and *E. coli* were the most common Gram-positive and Gram-negative bacteria, respectively. Therefore, *S. aureus* (ATCC-6538) and *E. coli* (ATCC-8739) were used to test the antibacterial property of the composite films. The samples were cut into circular films with a diameter of 10 mm, and sterilized with ultraviolet radiation for 30 min. The bacteria suspension (0.2 mL, 10^7^ cfu/mL) was evenly coated on the culture dishes containing nutrient agar, and dried at room temperature for 10 min. Then, the sterilized films were stuck on the surface of the solid medium and cultured at 37 °C for 24 h. The areas of the inhibition zone were calculated according to Equation (5):

(5) $$A=\frac{\pi \times (D^{2}-d^{2})}{4},$$

where *A* is the areas of the inhibition zone in mm^2^; D is the diameter of the inhibition zone in mm; and d is diameter of the films in mm.

### 3.7. FTIR Analysis

The interactions between chitosan and agarose were studied by FTIR spectroscopy (Spectrum 100, PerkinElmer, Waltham, MA, USA). The films were applied directly onto the attenuated total reflection (ATR) cell. The spectra were produced with a wave number range from 4000 to 450 cm^−1^ at a resolution of 4 cm^−1^ over 16 cumulative scans.

### 3.8. Morphology Studies

The morphology of the selected samples was observed by a DSC-TX10 digital camera (SONY Corporation, Tokyo, Japan). Scanning electron microscopy (SEM) was also carried out on a SEM JSM-6330F (JEOL Corporation, Tokyo, Japan). The sample was pre-treated by coating with an ultra-thin gold before SEM measurement.

## 4. Conclusions

Several chitosan–agarose composite films were prepared by blending chitosan and agarose together in different ratios. The physical-chemical-mechanical properties of the composite films were characterized, and the results demonstrated that the composite films, especially the composite film with an agarose mass concentration of 60%, exhibited significantly improved performance in the areas of tensile strength, elongation-at-break, water vapor transmission rate, water uptake capacity, and antibacterial activity when compared with pure chitosan film. FTIR analysis, optical microscopy, and SEM characterized the chemical structures of these films, indicating hydrogen bonding interactions and good micro-pore structures. The composite films, especially the composite film with an agarose mass concentration of 60%, exhibit excellent potential to be used in food packaging and biomedical materials.

## Figures and Tables

**Table 1:** The antibacterial effects of the composite films with different agarose contents against *S. aureus* and *E. coli*.

Films with Agarose Mass Concentrations (%)	Areas of the Antibacterial Zone (A, mm^2^)	
	*S. aureus*	*E. coli*
0	89.29 ± 1.23	83.83 ± 0.95
20	78.89 ± 0.89	51.52 ± 2.11
40	71.64 ± 2.06	37.38 ± 1.58
60	70.56 ± 1.55	30.43 ± 0.73
80	22.09 ± 1.17	13.91 ± 1.35
